# Editorial: Cellular reprogramming in development, disease, and design of cell-replacement therapies

**DOI:** 10.3389/fcell.2023.1260021

**Published:** 2023-07-31

**Authors:** Zoë D. Burke, Cressida Lyon, Macarena Perán

**Affiliations:** ^1^ Department of Life Sciences, University of Bath, Bath, United Kingdom; ^2^ Department of Health Sciences, University of Jaén, Jaén, Spain

**Keywords:** differentiation, transdifferentiation, reprogramming, organoids, cell therapy, development

Cellular reprogramming is the process whereby one cell type can be converted to another, this includes directed differentiation, induced pluripotency and transdifferentiation. Understanding the mechanisms underlying normal cell and tissue development has provided insight to how these cell-type conversions arise and enabled the development of potential autologous cell-replacement therapies.

The goal of this Research Topic was to highlight novel and promising advances in the field of cellular reprogramming and differentiation that have potential utility in the design and development of cell replacement therapies. The Research Topic includes four articles that shed light on different aspects of regenerative medicine and cellular reprogramming, including kidney development and disease, osteoblast development, degenerative conditions and novel therapeutic targets for cardiovascular disease. These studies provide valuable insights into the underlying mechanisms of these processes and present potential avenues for future research and clinical applications.

The brief research report by Moretto Rodrigues et al. investigated the role of triiodothyronine (T3) in proliferation, differentiation and maintenance of osteoblasts. Using osteoblast-like cells differentiated from human adipose-derived mesenchymal stem cells, the authors show that T3 adversely affected osteoblast development by hindering crucial signaling pathways involved in bone metabolism. This study highlights the need for further research to elucidate the mechanisms of T3 action and its potential implications the pathophysiology of bone disease.

The latest advances into the potential of directed differentiation and transdifferentiation for cell-based therapies for the treatment of diverse diseases, including type I diabetes, myocardial infarction, neurodegenerative diseases and liver fibrosis, were reviewed by Kuang et al. For each disease, the authors highlight recent progress in the production of functional cell types and provide an overview of the starting cell types, factors and methods used in cellular reprogramming approaches. By discussing the underlying mechanisms of reprogramming and presenting recent progress in diverse functional cell types, the authors lay the foundation for future investigations and highlight the potential general principles governing reprogramming.

Safi et al. provide a comprehensive review on the development of kidney organoids derived from human pluripotent stem cells (hPSCs). These organoids provide a platform for studying kidney morphogenesis, renal differentiation and disease modelling. The authors describe the interplay of developmental pathways and discuss how CRISPR/Cas9 technology has enabled the recapitulation and correction of cellular phenotypes associated with renal disease. The limitations of kidney organoid technology are acknowledged, and the potential for bioengineering solutions to enhance standardization is discussed.

The merits and pitfalls of current interventions for cardiovascular disease were debated by Barungi et al. Currently, surgical intervention for cardiovascular disease relies on coronary artery bypass grafting or stent placement. However, immune responses and restenosis (a re-thickening of the vessel following surgery) frequently thwart these approaches. The authors highlight the capacity of harnessing technological advances, such as electrospinning and bioprinting, to generate cellularized vascular tissue engineered vascular grafts. Key aspects in the production of such grafts are discussed, including cell sources, and coating with therapeutically designed nanoparticles designed to improve biocompatibility.

The findings presented in these articles highlight the ongoing efforts in the scientific community and point towards promising future directions for biomedical research and clinical applications. By expanding our knowledge in these areas, researchers can pave the way for improved diagnostics, treatments, and regenerative medicine approaches.

